# Healthcare utilization trends in adults with asthma or COPD during the first year of COVID-19 pandemic in comparison to pre-pandemic: A population-based study

**DOI:** 10.1371/journal.pone.0316553

**Published:** 2025-03-06

**Authors:** Tetyana Kendzerska, Michael Pugliese, Douglas Manuel, Mohsen Sadatsafavi, Marcus Povitz, Therese A Stukel, Teresa To, Shawn D. Aaron, Sunita Mulpuru, Melanie Chin, Claire E. Kendall, Kednapa Thavorn, Andrea S. Gershon

**Affiliations:** 1 The Ottawa Hospital Research Institute, Ottawa, Ontario, Canada; 2 Department of Medicine, Faculty of Medicine, University of Ottawa, Ontario, Canada; 3 ICES, Ontario, Canada; 4 School of Epidemiology and Public Health, Faculty of Medicine, University of Ottawa, Ontario, Canada; 5 Respiratory Evaluation Sciences Program, Faculty of Pharmaceutical Sciences, The University of British Columbia, Vancouver, British Columbia, Canada; 6 Department of Medicine, Cumming School of Medicine, University of Calgary, Calgary, Alberta, Canada; 7 Sunnybrook Research Institute, Sunnybrook Health Sciences Centre, Toronto, Ontario, Canada; 8 Dalla Lana School of Public Health, University of Toronto, Toronto, Ontario, Canada; 9 Research Institute, The Hospital of Sick Children, Toronto, Ontario, Canada; 10 Bruyère Health Research Institute, Ottawa, Ontario, Canada; 11 The Department of Family Medicine, University of Ottawa, Ottawa, Ontario, Canada; 12 Department of Medicine, University of Toronto, Ontario, Canada; Universitair Kinderziekenhuis Koningin Fabiola: Hopital Universitaire des Enfants Reine Fabiola, BELGIUM

## Abstract

**Objectives:**

To assess how changes in outpatient services during the first year of the COVID-19 pandemic were related to acute healthcare use (emergency department or hospitalizations) for individuals with asthma or chronic obstructive pulmonary disease (COPD).

**Methods:**

We conducted an observational study using health administrative data in Ontario (Canada) from January 2016 to March 2021 on all adults with diagnosed asthma or COPD. We used monthly time series auto-regressive integrated moving-average (ARIMA) and pre-pandemic monthly rates (January 2016 to February 2020) to calculate projected rates (i.e., a pandemic had not occurred) during the pandemic (March 2020 to March 2021), and Quasi-Poisson models with two-way interaction to estimate crude and adjusted rate ratios.

**Results:**

In the first pandemic year, in individuals with asthma or COPD, outpatient visit rates started lower than projected (Mar-May 2020), returned to projected in the middle of the year (Jun-Aug 2020) and then rose to higher than projected between Sep 2020 and Mar 2021: observed rates of 80,293 per 100,000 persons vs. projected 74,192 (95% CI: 68,926-79,868) in individuals with asthma, and 92,651 vs. projected 85,871 (95% CI: 79,975-92,207) in individuals with COPD. Acute care rates remained below projected during the first pandemic year. While pulmonary function test (PFT) rates remained below projected during the first pandemic year, in both populations, a decrease in acute care visits during the pandemic, compared to pre-pandemic, was noted during months with the highest PFT rates (interaction p-values < 0.0001).

**Conclusions:**

Despite asthma and COPD being ambulatory-care sensitive conditions, lower rates of outpatient visits during the beginning of the pandemic were not associated with increased rates of acute care use. Lower PFT rates were associated with higher acute care visit rates, suggesting that access to PFT during pandemic is likely important for individuals with asthma or COPD.

## Introduction

Asthma and chronic obstructive pulmonary disease (COPD) are the most prevalent chronic respiratory diseases and remain a leading cause of mortality and disability worldwide [1] and are associated with significant healthcare utilization-related costs.[2] They are also ambulatory-care-sensitive conditions, meaning that acute care use (hospitalizations or emergency department [ED] visits) for those conditions could be prevented or reduced by appropriate ambulatory care, which refers to medical care provided in outpatient settings.[3, 4] This includes pulmonary function testing (PFT), which is used to diagnose and guide the management of asthma and COPD.[5]

In Ontario (Canada), for individuals with asthma 16 years and older, Ontario Health Quality Standards recommend a structured assessment, including spirometry as clinically needed, at least once per year to determine asthma control and reasons for poor control.[6] Adults who meet criteria for severe asthma or have other indications are referred to specialized asthma care. Adults who have had an ED visit or been hospitalized for an asthma exacerbation should have a follow-up assessment within 2 to 7 days post-discharge.[6] For adults with COPD, Health Quality Standards recommend a comprehensive assessment at least once per year.[7] Those who have been hospitalized due to a COPD exacerbation should have a follow-up assessment within 7 days post-discharge. Additionally, spirometry should be conducted to reassess airflow limitation severity whenever there is a change in the individual’s health status.[7] The Global Initiative for Chronic Obstructive Lung Disease (GOLD) guidelines, also followed in Canada, recommend spirometry at least once a year to identify individuals with COPD who are declining quickly.[8]

During the COVID-19 pandemic, there was limited in person ambulatory care, particularly at the beginning, attributed to stringent quarantine policies.[9, 10] Virtual care took its place. Likewise, PFT access was reduced because strict public health measures were implemented for aerosolizing procedures to limit COVID-19 transmission.[11] Despite this, surges in asthma and COPD hospitalizations and ED visits were not noted [9,12,13].

Specifically, at the beginning of the pandemic, the Canadian Thoracic Society (CTS) released a position statement for individuals with asthma [14] recommending continuing maintenance and exacerbation management for asthma following current asthma treatment guidelines. It has also been suggested that all patients with asthma should follow current public health advisories concerning the indications for physical distancing and isolation. While individuals with mild-moderate asthma were suggested to work from home if feasible, individuals with severe asthma were recommended to remain off work, regardless, if needed for medical reasons, until physical distancing is no longer necessary. Similarly, the CTS suggested individuals with COPD stay at home as much as possible and follow current public health advisories when they must leave home.[15] Although in-person programs were closed until further notice, self-management and pulmonary rehabilitation counseling had been recommended to be done remotely. The usual maintenance and exacerbation management for COPD had been recommended.

With ambulatory-care-sensitive conditions, such as asthma or COPD, the disruption in ambulatory care would be expected to result in an increase in hospitalizations for exacerbations.[16] As such, a decrease in both hospitalizations and outpatient care requires further scrutiny. Initially, low hospitalization rates were attributed to fewer circulating respiratory viruses and less air pollution to trigger exacerbations, and people not wanting to attend hospitals because they feared being infected with COVID-19 [9,17–20], but this was not necessarily the situation as things opened up.

Previous studies of health services use during the pandemic have only examined the initial months of the pandemic when significant lockdowns and fear were at their height.[9] They also examined either acute or outpatient health services use without considering how they influenced each other and/or only considered smaller, non-representative populations.[9] Thus, little is known about the relationship between outpatient, particularly virtual care, and acute health services use after the initial few months of the pandemic in large, representative asthma and COPD populations. Similarly, there is little know of the association between trends in outpatient PFTs during the pandemic and acute care use.

To address this gap, we conducted a population-based study to describe trends in outpatient services, acute healthcare use, and health system costs associated with healthcare utilization in adults with known asthma or COPD in the first year of the pandemic. This information is important to our understanding of how we can prepare for care of people with these conditions, should there be another pandemic. We also investigated how changes in rates of outpatient care services (primary and specialist care visits, virtual care visits and PFT) were related to acute healthcare use (ED visits and hospitalizations). We hypothesized that (i) rates of both acute and ambulatory services would be different during the pandemic compared to similar periods pre-pandemic; (ii) changes in rates of acute services use during the pandemic compared to the pre-pandemic period would vary according to the rates of ambulatory care services; and (iii) the rates of virtual visits introduced during the pandemic would be related to rates of acute care.

## Methods

### Study design

We conducted a retrospective province-wide open-cohort study using provincial health administrative data of Ontario (population of 15 million), Canada, on all adults who had been diagnosed with asthma or COPD between January 2016 and March 2021 inclusive. We compared monthly rates of healthcare utilization in the first pandemic year (March 2020-March 2021) to rates in the similar pre-pandemic period (January 2016-December 2019). A one-year pandemic period was selected to understand trends after the initial impact of the pandemic.

### Ethical statement

ICES is a prescribed entity under Ontario’s Personal Health Information Protection Act (PHIPA). Section 45 of PHIPA authorizes ICES to collect personal health information, without consent, for the purpose of analysis or compiling statistical information with respect to the management of, evaluation or monitoring of the allocation of resources to or planning for all or part of the health system. Projects that use data collected by ICES under section 45 of PHIPA, and use no other data, are exempt from REB review. The use of the data in this project is authorized under section 45 and approved by ICES’ Privacy and Legal Office. Under the umbrella of the larger project (not only ICES-based), the current study was also approved by the Ottawa Health Science Network Research Ethics Board (OHSN-REB) (Protocol ID: 20200482-01H). On March 13, 2023, the data were accessed for research purposes.

All methods were carried out in accordance with relevant guidelines and regulations. The study datasets were linked using unique encoded identifiers and analyzed in the secure environment at ICES following Ontario privacy standards. Authors had no access to information that could identify individual participants during or after data collection.

### Data sources

ICES is a non-profit institution housing individual-level health administrative databases on publicly funded services, including outpatient and inpatient services and diagnostic testing in Ontario.[21] Canada provides “universal coverage for medically necessary health care services provided on the basis of need, rather than the ability to pay.[22] These databases housed at ICES are regularly updated and checked for accuracy (www.datadictionary.ices.on.ca/). [23, 24] The Registered Persons (RPDB) database contains basic demographics and vital statistics. The Ontario Health Insurance Plan (OHIP) database captures inpatient and outpatient visits and PFTs (but not results). The National Ambulatory Care Reporting System Database (NACRS) and The Discharge Abstract Database (DAD) record ED visits and hospitalizations, respectively, and the Canadian Census includes neighborhood income and rurality indicators. The Ontario Mental Health Reporting System (OMHRS) contains inpatient records for mental health and substance use. ICES databases were linked on an individual level using unique encoded identifiers. Authors had no access to information that could identify individual participants during or after data collection.

### Population and setting

We used repeated monthly sampling of all adults (18 years and older) with a pre-existing diagnosis of asthma or COPD living in Ontario at the beginning of each month between January 2016 – March 2021. We used previously validated definitions (S1 Table) for both asthma [25] or COPD diagnosis [26] and only included those with a healthcare encounter for their condition within the last 5 years. Repeated sampling allowed individuals to enter and leave the cohort as their diagnostic status, age, residence, and OHIP eligibility changed over time. Follow-up continued until March 31, 2021.

#### Pandemic timeframe definitions.

We considered March 17, 2020, as the start of the pandemic,[27] as on March 15, 2020, the Minister of Health in Ontario requested to reduce non-emergent clinical activities to release health system capacity. [28] Our study time frame depicted the first wave of the COVID-19 infection (March-May 2020), along with a phased reopening during a period of declining and then relatively low case and COVID-19 hospitalizations (June-September 2020),[29] followed by a second wave of increasing cases and COVID-19 hospitalizations starting in mid-September 2020 until March 2021. Observed and projected rates were visualized monthly to avoid arbitrarily chosen time periods. For tabulations, similarly to our previous study,[17] rates were reported and compared for four time periods: (i) January-February 2020 (early spread); (ii) March-May 2020 (first wave); (iii) June-August 2020 (summer lockdown relaxation); and (iv) September 2020-March 2021 (second wave).

### Outcomes

#### Primary objectives.

The ***primary outcomes*** were outpatient care and acute care use. Outcomes were expressed as monthly rates per 100,000 persons at-risk. We reported outpatient care use as (i) all-cause outpatient visits, (ii) primary care visits, (iii) specialist visits, (iv) virtual visits, and (v) PFTs. We reported acute care use as (i) hospitalizations and (ii) ED visits.

Health system cost (***secondary***) were calculated from a public payer’s perspective using the hybrid costing methods developed specifically for Ontario health administrative data (see details in the **Data Supplement in** S1 Text).[30] All costs were standardized using health sector-specific consumer price indices to their equivalent 2021 Canadian dollar value.[31] Costs were expressed as millions of Canadian dollars per month.

#### Secondary objectives.

We investigated the relationship between changes in the rates of outpatient healthcare services and acute health services rates since the pandemic compared to pre-pandemic. We also investigated the relationship between virtual visits and acute care services use during the pandemic.

#### Covariates (applicable to secondary objectives only).

Covariates were sex, age, residence location (rural vs. urban),[32] neighborhood income quintiles,[33–35] and mental health and problematic substance use status (based on inpatient and outpatient mental health and addictions-related health services).[17] Details on definitions are provided in S1 Table and S2 Text.

### Statistical analyses

#### Primary objectives.

We created a 63-month time series from January 2016 to March 2021. Crude monthly outpatient and acute care use rates were calculated as the number of events per 100,000 persons at risk. Monthly costs were expressed in millions of Canadian dollars. We used auto-regressive integrated moving-average (ARIMA) models and pre-pandemic monthly rates (January 2016 to February 2020) to calculate projected outcome rates. Autoregressive (AR) and moving-average (MA) terms were fit to time series data to account for seasonality and other time series data to trends.[36] When fitted manually, the time series is differenced to create a linear series, and terms are added to the model based on visual interpretation of autocorrelation plots within which seasonal and non-seasonal AR and MA “signatures” are present. However, the number of ARIMA models to be run made manual fitting impractical, so, we used the automated model selection feature included in SAS software’s adaptation of the United States Census Bureau’s X-13ARIMA-SEATS program (X13) for ARIMA.[37–39] After selecting the best-fitting ARIMA model for each outcome, the model projected rates and 95% CIs for the 13 months following February 2020 (up to March 2021). Observed rates outside the 95% CIs were considered significantly different from projected.[9] More details on the ARIMA procedure are provided in S3 Text. We presented comparisons between observed and projected monthly rates both graphically as a time series and as mean rates across the four time periods in tabular form. All the above-mentioned analyses were done in the total population and stratified by sex and age (18-24, 25-34, 35-49, 50-64, 65 years and older) group.

The main analysis permitted the automated model procedure to select up to fourth-order non-seasonal terms and first-order seasonal terms to allow for maximum flexibility, while lessening the chances of overfitting the model. As a sensitivity analysis, we limited the automated model procedure to first-order terms for both the non-seasonal and seasonal term values and re-calculated projected values.

#### Secondary objective.

To investigate the hypothesized changing relationship between different healthcare services post-pandemic compared to pre-pandemic, we defined our exposure as a two-way statistical interaction between rates of outpatient healthcare services and time-period with acute care service rates as the outcome. We categorized monthly rates of outpatient healthcare services into quantiles. The time period was defined as a binary indicator: “pre-COVID” (January 2015 to February 2020) and “post-COVID.” The acute care service outcome was quantified as a count variable. Quasi-Poisson models were used to estimate crude and adjusted rate ratios (RRs).[40] We explored the two-way interaction using contrast statements to compare acute care rates post-COVID to pre-COVID within each quantile of outpatient care service use, adjusting for the covariates described above.

The above analysis was repeated with virtual visits as the exposure. However, we restricted the analysis of virtual visits to the pandemic period only because virtual visits pre-pandemic were extremely rare.

We performed all data analyses in SAS (version 9.4 using SAS Enterprise guide version 7.15.3) in the secure environment at ICES following Ontario privacy standards.

## Results

### Patient characteristics at the beginning of the pandemic

At the beginning of the pandemic, in March 2020, 620,572 adults had a physician diagnosis of asthma: 40.5% were male, 25.2% were 65 years and older, and 41.3% resided in low-income areas. During the same month, 458,224 adults had a physician diagnosis of COPD: 50.7% were male, 56.7% were 65 years and older, and 48.3% resided in low-income areas.

### Primary objectives

#### Outpatient healthcare services.

**Fig 1:**
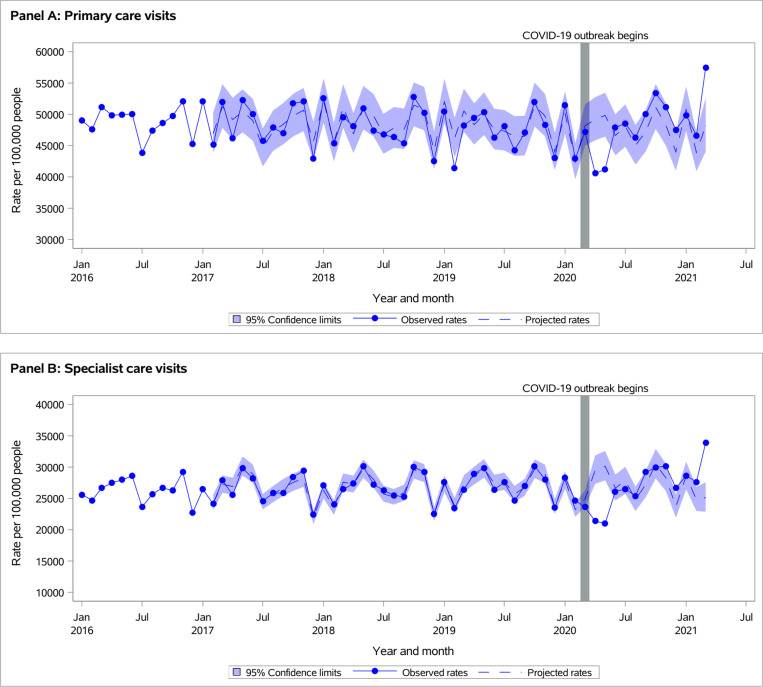
Observed versus projected monthly outpatient care service rates in adults with asthma per 100,000 persons at-risk: (A) Primary care visits, and (B) Specialist care visits.

**Fig 2:**
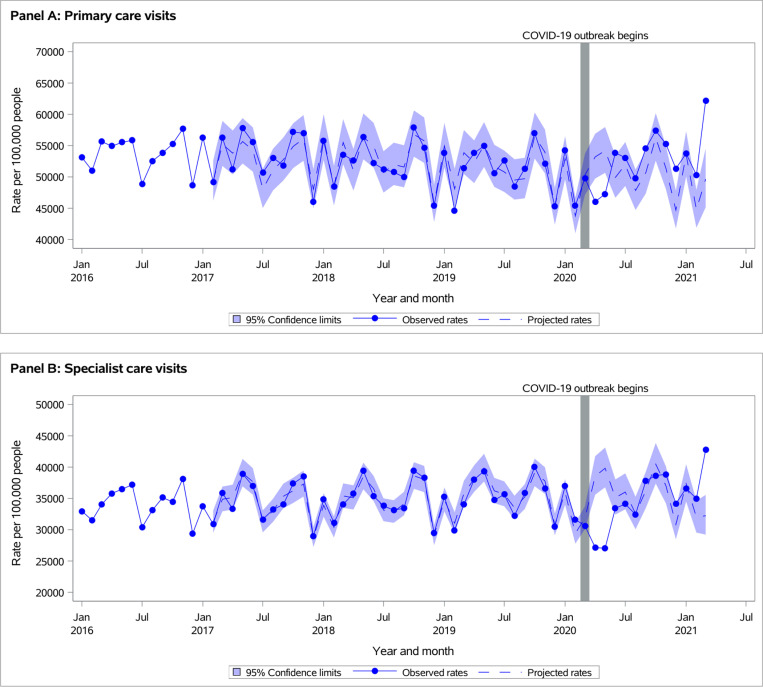
Observed versus projected monthly outpatient care service rates in adults with COPD per 100,000 persons at-risk: (A) Primary care visits, and (B) Specialist care visits.

In both populations of individuals with asthma or COPD, all-cause outpatient care visit rates were lower than projected during *March-May 2020*, returned to projected during *June-August 2020*, and were significantly higher than projected during *September 2020-March 2021* (Figs 1 and 2, S2-S3 Tables). During *September 2020-March 2021*, observed rates per 100,000 persons at risk of all outpatient visits were 80,293 vs. projected 74,192 (95% CI 68,926-79,868) for individuals with asthma and 92,651 vs. projected 85,871 (95% CI 79,975-92,207) for individuals with COPD population (S3 Table).

In both populations of individuals with asthma or COPD, PFT rates were below projected during the whole first year of the pandemic (S2-S3 Tables). During *September 2020-March 2021*, observed rates were 1,247 vs. 2,526 (95% CI 2,298-2,777) in individuals with asthma and 1,703 vs. projected 3,355 (95% CI 3,007-3,743) in individuals with COPD (S3 Table).

### Acute healthcare services

**Fig 3:**
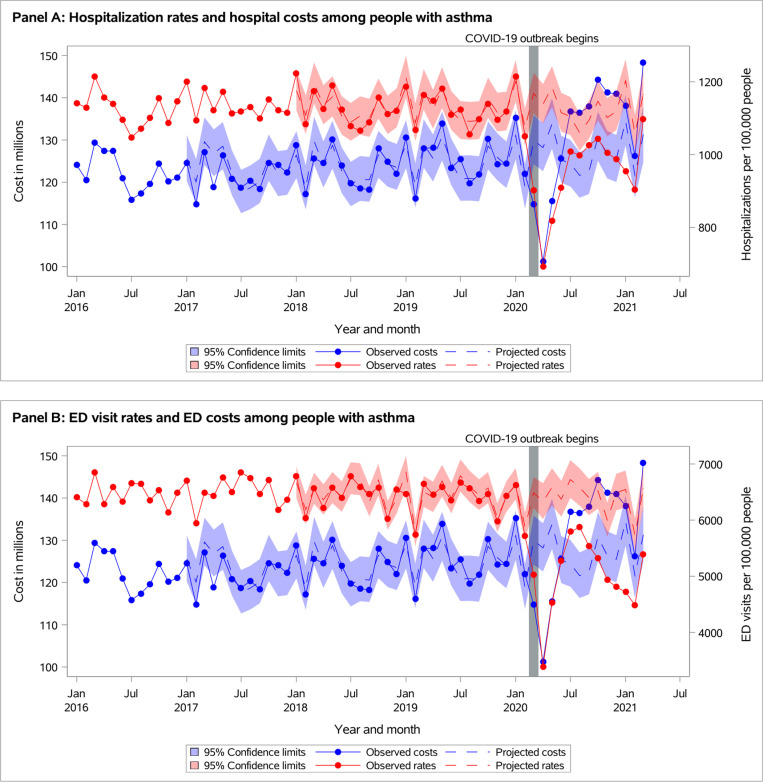
Observed versus projected monthly hospital costs and acute care visit rates in adults with asthma per 100,000 persons at-risk: (A) Hospitalizations, and (B) Emergency department (ED) visits.

**Fig 4:**
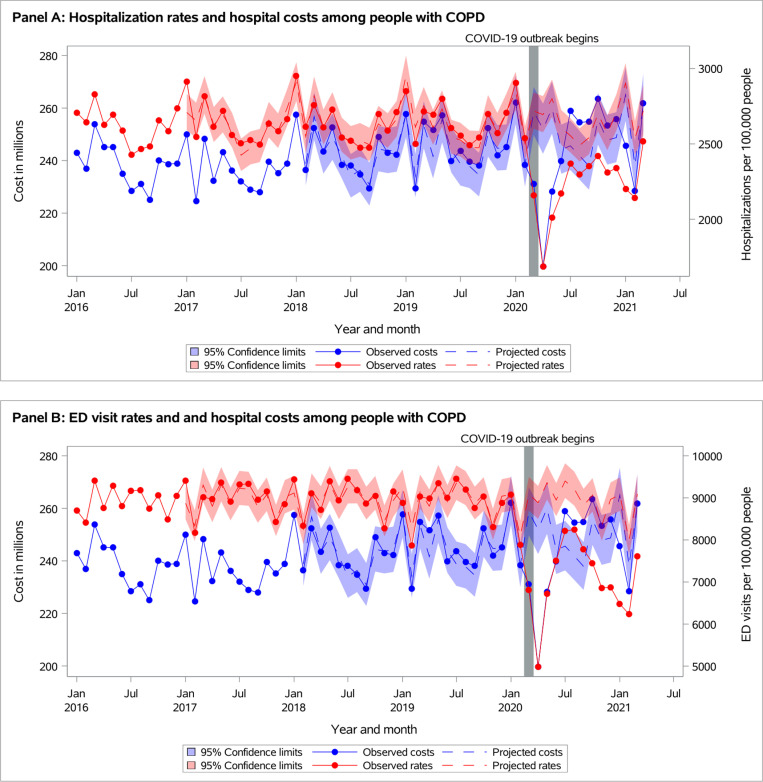
Observed versus projected monthly hospital costs and acute care visit rates in adults with COPD per 100,000 people at-risk: (A) Hospitalizations, and (B) Emergency department (ED) visits.

All-cause ED visit and hospitalization rates remained below projected during the first year of the pandemic in both populations (Figs 3 and 4, S2-S3 Tables). During *September 2020-March 2021*, observed hospitalization rates were 1,003 vs. projected 1,127 (95% CI 1,073-1,183) for individuals with asthma and 2,326 vs. 2,668 (95% CI 2,545-2,796) for individuals with COPD (S3 Table).

There was no significant variation by age or sex (S4-S5 Tables). Results of the sensitivity analyses were consistent with the main analysis (S6 Table).

#### Health system costs.

The observed total health system cost was significantly reduced during *March-May 2020* and then returned to the projected in both populations of individuals with asthma or COPD (Figs 3 and 4, S7 Table). Similarly, medication- and laboratory-related costs dropped at the beginning of the pandemic and then returned to the projected. In individuals with asthma, though the observed hospitalization cost reduced during *March-May 2020*, it increased above projected starting in June 2020 and remained above projected during *September 2020-March 2021*: $140 million vs. $128 (95% CI $122-$133) (Fig 3). In contrast, in individuals with COPD, the observed hospitalization cost returned to projected in June 2020 and remained as projected by the end of the year (Fig 4). In individuals with asthma, the observed physician-related cost returned to projected in June 2020 and remained as projected by the end of the year, while physician-related cost remained below projected by the end of the year in COPD population.

### Secondary objectives

#### Changes in healthcare service use post-pandemic.

Trends in changes between outpatient visit rates and acute healthcare use were similar for individuals with asthma or COPD (Table 1). Primary care visits and COVID-period interaction terms were significant for all-cause ED visits and hospitalizations (interaction p-values < 0.0001). This means that, in both populations, a decrease in the rate of ED visits during the pandemic, compared to pre-pandemic, was greater during those months with lower rates of primary care visits than those with higher rates. The same pattern was noted for all-cause hospitalizations in individuals with COPD. In individuals with asthma, the decrease in hospitalization rates during the pandemic compared to pre-pandemic was greater during months with higher rates of primary care visits.

**Table 1:** Adjusted rate ratios (aRR)^a^ comparing hospitalization and emergency department (ED) visit rates during the pandemic (March 2020 to February 2021) to the pre-pandemic period (March 2019 to February 2020) by quantiles of ambulatory service use visits.

Monthly rates of ambulatory healthcare services	All-cause ED visits	Interaction P value	All-cause Hospitalizations	Interaction P value
**aRR (95% CI)**	**aRR (95% CI)**
**All outpatient visits**				
***Asthma Population***				
Quantile I	**0.73 (0.71-0.75)**	<.0001	**0.88 (0.83-0.93)**	0.0006
Quantile II	**0.78 (0.77-0.79)**		**0.82 (0.80-0.84)**	
Quantile III	**0.81 (0.79-0.82)**		**0.87 (0.85-0.89)**	
***COPD Population***				
Quantile I	**0.80 (0.78-0.81)**	<.0001	**0.83 (0.81-0.85)**	<.0001
Quantile II	**0.77 (0.75-0.78)**		**0.80 (0.79-0.81)**	
Quantile III	**0.83 (0.82-0.85)**		**0.88 (0.86-0.90)**	
**Primary care visits**				
***Asthma Population***				
Quantile I	**0.75 (0.73-0.77)**	<.0001	**0.94 (0.88-0.99)**	<.0001
Quantile II	**0.77 (0.76-0.78)**		**0.80 (0.79-0.82)**	
Quantile III	**0.82 (0.80-0.83)**		**0.86 (0.84-0.89)**	
***COPD Population***				
Quantile I	**0.79 (0.77-0.81)**	<.0001	**0.84 (0.81-0.86)**	<.0001
Quantile II	**0.76 (0.75-0.78)**		**0.80 (0.79-0.81)**	
Quantile III	**0.82 (0.81-0.84)**		**0.88 (0.86-0.90)**	
**Other specialist care visits**				
***Asthma Population***				
Quantile I	**0.74 (0.72-0.76)**	<.0001	**0.81 (0.76-0.87)**	0.3846
Quantile II	**0.77 (0.76-0.78)**		**0.83 (0.81-0.85)**	
Quantile III	**0.79 (0.78-0.81)**		**0.84 (0.83-0.86)**	
***COPD Population***				
Quantile I	**0.80 (0.76-0.84)**	0.4828	**0.87 (0.82-0.93)**	0.0047
Quantile II	**0.79 (0.78-0.81)**		**0.82 (0.80-0.83)**	
Quantile III	**0.81 (0.79-0.82)**		**0.85 (0.84-0.86)**	
**Pulmonary Function Test (PFT)**				
***Asthma Population***				
Quantile I	**0.61 (0.51-0.72)**	<.0001	0.88 (0.61-1.28)	<.0001
Quantile II	**0.87 (0.84-0.89)**		**1.22 (1.14-1.30)**	
Quantile III	**0.79 (0.78-0.81)**		**0.87 (0.85-0.88)**	
***COPD Population***				
Quantile I	0.74 (0.56-1.00)	<.0001	0.78 (0.57-1.07)	<.0001
Quantile II	1.04 (0.99-1.10)		**1.13 (1.05-1.21)**	
Quantile III	**0.80 (0.79-0.82)**		**0.86 (0.84-0.87)**	

^a^Reference group: pre-COVID period.

In bold: statistically significant

All models were adjusted for sex, age, rurality, income, and mental health and substance use status.

aRR, adjusted rate ratios; CI, confidence intervals; ED, emergency department

In both populations of individuals with asthma or COPD, a significant (p < 0.0001) decrease in ED visit and hospitalization rates during the pandemic, compared to pre-pandemic, was noted during months with the highest rates of PFTs (interaction p-values < 0.0001).

### Virtual visits and acute care service use during the pandemic

In individuals with asthma, restricted to the pandemic only and compared to months with the lowest rates of virtual care visits (Quantile I), months with the highest rates were associated with decreased ED visits and hospitalization rates (p-values < 0.001) (Table 2). In individuals with COPD, compared to months with the lowest rates of virtual care visits, months with higher rates were associated with increased ED visit rates for all except the highest quantile (p-values < 0.0001); months with the highest rates of virtual care visits (Quantile V) were associated with decreased hospitalization rates (p = 0.01) (Table 2).

**Table 2:** Adjusted rate ratios (aRR)^a^ comparing hospitalization and emergency department (ED) visit rates during the pandemic by quantiles of virtual care visit rates since the beginning of the pandemic.

Quantiles for monthly rates^b^	Asthma Population				COPD Population		
	Outcome rate per 100,000 people at risk	aRR (95% CI)	P Value	Outcome rate per 100,000 people at risk		aRR (95% CI)	P Value
	**ED visits**						
Quantile I	12,177.84	Reference group		10,351.51		Reference group	
Quantile II	6,358.73	**1.15 (1.10-1.20)**	<.0001	10,342.32		**1.21 (1.15-1.28)**	<.0001
Quantile III	4,802.27	**1.11 (1.06-1.16)**	<.0001	7,516.32		**1.21 (1.15-1.28)**	<.0001
Quantile IV	6,240.29	**1.10 (1.05-1.16)**	<.0001	8,531.30		**1.19 (1.13-1.26)**	<.0001
Quantile V	7,732.46	**0.92 (0.87-0.97)**	0.0009	13,926.02		1.05 (0.99-1.11)	0.1073
	**Hospitalizations**						
Quantile I	2,217.45	Reference group		4,631.65		Reference group	
Quantile II	1,428.60	**1.11 (1.05-1.18)**	0.0003	3,626.53		**1.15 (1.10-1.20)**	<.0001
Quantile III	903.91	1.01 (0.95-1.07)	0.7750	2,125.19		**1.13 (1.08-1.18)**	<.0001
Quantile IV	727.98	0.94 (0.89-1.01)	0.0739	1,799.30		1.04 (0.99-1.09)	0.0913
Quantile V	668.38	**0.76 (0.71-0.82)**	<.0001	2,357.41		**0.94 (0.89-0.99)**	0.0131

^a^All models were adjusted for sex, age, rurality, income, and mental health and substance use status.

^b^Quantiles for monthly rate of virtual care visits: higher number =  month with higher usage rates.

In bold: statistically significant.

aRR, adjusted rate ratios; CI, confidence intervals; ED, emergency department

## Discussion

We conducted a population-based, retrospective study to describe healthcare utilization in a large, complete population of adults with asthma or COPD and found that, after the initial three months of the pandemic, ED visits and hospitalization remained low while outpatient care visits recovered to pre-pandemic or higher levels by the end of the first pandemic year (March 2021). Lower outpatient care visit rates were not associated with increased acute care use. We also found that PFT rates remained below the projected rates during the first pandemic year and that these lower rates were associated with higher ED visits and hospitalizations. Finally, more virtual care visits were associated with decreased acute care use in individuals with asthma. To the best of our knowledge, this is the first comprehensive description of the relationship between outpatient and acute services use during the first years of the pandemic. This information might help to understand trends in acute healthcare use for individuals with asthma or COPD in response to a reduction in outpatient care or substitution by virtual care. This has relevance to future planning for health services in the event of limitations on ambulatory care services.

Our findings of reduced outpatient care visit rates and PFTs at the beginning of the pandemic are consistent with other studies that demonstrated disrupted outpatient care and diagnostics services in individuals with COPD or asthma.[41] A survey conducted in British Columbia (Canada) demonstrated that among healthcare services for individuals with asthma or COPD, specialty care services were most frequently reported as disrupted, while primary and home care and diagnostic tests were least disrupted.[41] A study conducted in Beijing at the beginning of the pandemic reported that approximately 30% of individuals with COPD experienced worsening respiratory symptoms, but most did not seek medical help due to fear of cross-infection.[42]

A significant reduction in COPD hospitalizations during the pandemic was also observed in other studies.[20] There are several explanations for reduced ED visits and hospitalization for individuals with asthma or COPD during the first pandemic year, including: (i) fear of COVID-19 infection,[42] especially given that individuals with airway disease may be at higher risk of developing adverse COVID-19-related outcomes;[43, 44] (ii) a lack of/limited screening or diagnostic capabilities; (iii) a global reduction in the prevalence and transmission of many seasonal respiratory viruses;[45, 46] (iv) reduced air pollution [47] due to stay-at-home orders and remote work; (v) improved ability to manage their disease with more time in lockdown (i.e., increased adherence to medications),[48] and (vi) a transition into alternative forms of care, including virtual care.

A reduction in PFT rates during the pandemic could be explained by PFT laboratories shutting down or reducing services to reduce virus transmission by aerosolizing procedures.[11,49] This likely influenced care of individuals with COPD or asthma and was associated with the increased ED visits and hospitalizations observed. Modifications to PFT laboratory protocols or the use of alternate biomarkers present an opportunity to mitigate the ongoing risk of transmitting the SARS-CoV-2 virus and other emerging pathogens.[49]

Our study provides a glimpse into the effectiveness of virtual care. While virtual care is usually considered inferior to in-person care, especially for respiratory diseases, it may benefit some populations,[50] as evidenced by it being associated with possible decreased hospitalizations in individuals with asthma or COPD.

The COVID-19 pandemic had a multifaceted impact on healthcare and its costs in individuals with COPD or asthma. Healthcare utilization and costs decreased during the first few months of the pandemic as patients avoided seeking medical care due to concerns about virus exposure. Non-urgent appointments, diagnostic tests, and elective procedures were also pushed back. As the situation stabilized, healthcare costs rose to pre-pandemic levels, likely due to patients catching up on missed appointments.

In both individuals with asthma or COPD, hospitalization rates remained lower than projected during the first year of the pandemic. Despite this, hospital costs exceeded (asthma) or rose to levels comparable to (COPD) projected estimates. This could be attributed to changes in hospital services, higher labour costs, supply chain disruptions, new protocols and procedures, and personal protective equipment, testing, and screening costs.[51, 52]

Our study has several strengths, including its open cohort sampling approach, monthly analysis, and the ARIMA analytic used to consider baseline trends before the pandemic for more accurate analysis. It also had limitations. First, we were not able to adjust for the complexity of outpatient and acute care and to delve into cost-related findings further. Information on symptoms and the results of PFT were also not available. Second, trends in COVID-19 transmission in the community likely confounded the PFT findings. However, having a PFT could influence care, for example, lead to better diagnosis or disease control, impacting ED visits or hospitalizations.[53] Investigating the role of PFT during the pandemic is an important area for future studies. In addition, this study focused on healthcare service impacts rather than other effects of aggressive infection control measures in the community, which could confound our findings, such as the reduction in air pollution and non-COVID respiratory infections. Third, our results are likely generalizable to Canada; however, they need to be repeated for other settings. Fourth, although our study focused only on the first year of the pandemic, this year was critical for individuals with chronic conditions, specifically airway diseases, given the significant restrictions in health care services in this population, the rapid shift from in-person to virtual care and possible association with the risk of COVID-19 infection and its complications. Future studies are also required to describe more recent trends in healthcare utilization since the pandemic in individuals with asthma or COPD as well as determining if care provision met standards after the pandemic.

## Conclusion

In this population-based retrospective study of adults with asthma or COPD, we demonstrated lower rates of outpatient care were not associated with increased acute care use. ED visits and hospitalizations remained low for the first year of the pandemic, which could be at least partially explained by implementing aggressive infection control measures in the community to prevent COVID-19 with off-target effects which benefited patients with airway disease, protecting them from respiratory infections in general, and thus resulted in fewer ED visits and hospitalizations. Our results suggested that access to PFT during pandemic is likely important for individuals with COPD or asthma, and virtual care could be a reasonable substitute for in-person visits if needed, especially in individuals with asthma, which required confirmation in future studies.

## Guarantor statement

Together, Tetyana Kendzerska and Michael Pugliese had full access to all the data in the study and took responsibility for the integrity of the data and accuracy of the data analysis. They affirm that the manuscript is an honest, accurate, and transparent account of the study being reported; no important aspects of the study have been omitted; any discrepancies from the study as planned have been explained. All authors had full access to statistical reports and tables.

## Supporting information

S1 Text Details on the cost calculations from health administrative data.
(DOCX)

S2 Text Details on mental health status definition.
(DOCX)

S3 Text Details on the ARIMA procedure.
(DOCX)

S1 Table Definitions to derive the population of interest and variables at baseline from health administrative databases.
(DOCX)

S2 Table (A-B). Monthly crude rates, crude rate ratios (RR) and 95% confidence intervals (CI) for all-cause outpatient, inpatient visits, and pulmonary function tests (PFT) in adults with a pre-existing physician diagnosis of asthma or COPD during the first year of the pandemic compared to pre-pandemic.
(DOCX)

S3 Table (A-B). Observed and projected monthly rates and 95% confidence intervals (CI) estimated by ARIMA Models for all-cause hospitalizations, emergency department (ED) and outpatient visits in adults with a pre-existing physician diagnosis of asthma or COPD: rates were calculated as the number of events per 100,000 people at risk. Similar periods in previous years (2016-2019) were used to calculate projected rates.
(DOCX)

S4 Table (A-D). Observed and projected monthly rates and 95% confidence intervals (CI) estimated by ARIMA Models for all-cause hospitalizations, emergency department (ED) and outpatient visits in adults with a pre-existing physician diagnosis of asthma (total and stratified by sex and age): rates were calculated as the number of events per 100,000 people at risk. Similar periods in previous years (2016-2019) were used to calculate projected rates.
(DOCX)

S5 Table (A-D). Observed and projected monthly rates and 95% confidence intervals (CI) estimated by ARIMA Models for all-cause hospitalizations, emergency department (ED) and outpatient visits in adults with a pre-existing physician diagnosis of COPD (total and stratified by sex and age): rates were calculated as the number of events per 100,000 people at risk. Similar periods in previous years (2016-2019) were used to calculate projected rates.
(DOCX)

S6 Table (A-B). A sensitivity analysis: Observed and projected monthly rates and 95% confidence intervals (CI) estimated by ARIMA Models for all-cause hospitalizations, emergency department (ED) and outpatient visits in adults with a pre-existing physician diagnosis of asthma or COPD: rates were calculated as the number of events per 100,000 people at risk. Similar periods in previous years (2016-2019) were used to calculate projected rates. As a sensitivity analysis, we limited the automated model procedure to first-order terms for both the non-seasonal and seasonal term values and re-calculated projected values.
(DOCX)

S7 Table Observed and projected costs (total and by subgroups) estimated by ARIMA Models for costs in individuals with a pre-existing physician diagnosis of asthma or COPD: in millions, 2021 adjusted dollars.
Similar periods in previous years (2016-2019) were used to calculate projected costs.
(DOCX)
