# Supplementary material for: Healthcare utilization trends in adults with asthma or COPD during the first year of COVID-19 pandemic in comparison to pre-pandemic: A population-based study
Source: PLoS One. 2025 Mar 6;20(3):e0316553. doi: 10.1371/journal.pone.0316553 (PMC11884700; doi:10.1371/journal.pone.0316553)
Supplement: S1 Text — (DOCX) [file pone.0316553.s001.docx]

**S1 Text.** **Details on the health system cost calculations from health administrative data.**

The standardized previously developed approach (GETCOST macro) was used to determine individual-level health system costs from a public payer's perspective.[1] Costs included hospital admissions, ED visits, same-day surgery visits, cancer centre treatments and visits, dialysis, other outpatient care (e.g., laboratory services), physician costs, homecare and long-term services, and prescription medications (covered by the Ontario Drug Benefit program for eligible individuals). All costs were standardized using health sector-specific consumer price indices to their equivalent 2021 Canadian dollar value.[2]

**S-References**

1. Wodchis WP, Bushmeneva K, Nikitovic M, McKillop I. Guidelines on Person-Level Costing Using Administrative Databases in Ontario. Toronto: Health System Performance Research Network2013.

2. Consumer price index portal Statistics Canada [May 4, 2023]. Available from: <https://www.statcan.gc.ca/en/subjects-start/prices_and_price_indexes/consumer_price_indexes>.
