# Supplementary material for: Healthcare utilization trends in adults with asthma or COPD during the first year of COVID-19 pandemic in comparison to pre-pandemic: A population-based study
Source: PLoS One. 2025 Mar 6;20(3):e0316553. doi: 10.1371/journal.pone.0316553 (PMC11884700; doi:10.1371/journal.pone.0316553)
Supplement: S2 Text — (DOCX) [file pone.0316553.s002.docx]

**S2 Text.** **Details on mental health status definition.**

Mental health status was based on a previously developed mental health and addictions-related services classification algorithms for outpatient visits (the Ontario Health Insurance Plan [OHIP]), ED visits (the National Ambulatory Care Reporting System [NACRS]) and inpatient hospitalizations (the Discharge Abstract Database [DAD] and the Ontario Mental Health Reporting System [OMHRS]) for mental health and addictions-related complaints and ICES dementia cohort definition.

| **Definition of mental health condition at baseline (Y/N)** | One or more mental health hospitalization/ED visit/outpatient visits within the last year OR met dementia definition | |
| --- | --- | --- |
| **Hospitalizations/ED visits (from CIHI-DAD, NACRS, and OMHRS): Y/N** | | |
| **Hospitalizations/ED visits** | **ICD-10-CA (DAD/NACRS)** | **DSM or provisional diagnoses* (OMHRS)** |
| Overall, any mental health disorder or addiction | Primary diagnosis at discharge equals F06−F99 (which excludes dementia), or secondary diagnoses fields equal X60−X84, Y10−Y19, Y28 when primary diagnosis is not F06−F99 | **DSM IV (For visits before April 1^st^, 2016):**   - AXIS1_DSM4CODE_DISCH1 = Any OMHRS diagnosis (includes missing; excludes 290.x, 294.x). - Exclude if AXIS1_DSM4CODE_DISCH1 missing and PROVDX_DSM4CODE_ADM1 =2   **DSM V (visits April 1^st^, 2016, and after):**   - DSM5CODE_DISCH1 = Any OMHRS (includes missing; excludes 290.x, 294.0x-294.7x, 294.9x). - Exclude if DSM5CODE_DISCH1 missing and PROVDX_DSM5CODE_ADM1 =17 |
| **Outpatient visits (from OHIP): Y/N** | | |
| **Outpatient visits** | **Algorithm** | **​Qualifying diagnoses codes (DXCODE)** |
| Overall, any mental health disorder or addiction | Psychiatrist [SPEC=19] and outpatient (LOCATION: O, L, H) and non-lab service [substr(FEECODE,1,1) ne 'G'] *OR*  FP/GP [SPEC=00] and MHA diagnosis code [DXCODE] and outpatient (LOCATION: O, L, H) and non-lab service [substr(FEECODE,1,1) ne 'G'] | ​**Psychotic Disorders**   - 295 Schizophrenia - 296 Manic-depressive psychoses, involutional melancholia - 297 Other paranoid states - 298 Other psychoses   **Non-Psychotic Disorders**   - 300 Anxiety neurosis, hysteria, neurasthenia, obsessive-compulsive neurosis, reactive depression - 301 Personality disorders - 302 Sexual deviations - 306 Psychosomatic illness - 309 Adjustment reaction - 311 Depressive disorder   **Substance Use Disorders**   - 303 Alcoholism - 304 Drug dependence   **Social Problems**   - 897 Economic problems - 898 Marital difficulties - 899 Parent-child problems - 900 Problems with aged parents or in-laws - 901 Family disruption/divorce - 902 Education problems - 904 Social maladjustment - 905 Occupational problems - 906 Legal problems - 909 Other problems of social adjustment |
| **Dementia: Y/N** | | |
| Met dementia definition | A person aged 40 to 110 years old is identified with dementia if s/he meets one of the following criteria:   - The person had at least 3 OHIP claims with a dementia diagnosis recorded which were each at least 30 days apart in a 2-year period, or - The person had at least one hospitalization or same day surgery with a dementia diagnosis recorded, or - The person had at least one ODB claim with a dementia medication (SUBCLNAM= CHOLINESTERASE INHIBITORS) dispensed | |

DAD, the Discharge Abstract Database; DSM, the Diagnostic and Statistical Manual; ED, emergency department; NACRS, the National Ambulatory Care Reporting System; ODB, the Ontario Drug Benefit; OHIP, the Ontario Health Insurance Plan; OMHRS, the Ontario Mental Health Reporting System.
