# Supplementary material for: Healthcare utilization trends in adults with asthma or COPD during the first year of COVID-19 pandemic in comparison to pre-pandemic: A population-based study
Source: PLoS One. 2025 Mar 6;20(3):e0316553. doi: 10.1371/journal.pone.0316553 (PMC11884700; doi:10.1371/journal.pone.0316553)
Supplement: S3 Text — (DOCX) [file pone.0316553.s003.docx]

**S3 Text. Details on the ARIMA procedure.**

We identified healthcare usage using CIHI's Discharge Abstract Database (DAD) for inpatient acute care, CIHI's National Ambulatory Care Reporting System (NACRS) for emergency department visits, and the Ontario Health Insurance Plan (OHIP) database for outpatient visits. Monthly rates are calculated as the number of events per 100,000 person-days. A person accrued person-days in a month until they either died or lost their OHIP eligibility.

We used autoregressive integrated moving average (ARIMA) models to calculate projected rates. ARIMA models regress an outcome on its past values, fitting autoregressive (AR) and moving-average (MA) terms to a time series once it has been made stationary, often through differencing (integration). Seasonal terms can be added to the model to account for the correlation structure of regular seasonal patterns in a time series. These models are sometimes referred to as seasonal ARIMA models (SARIMA).

AR and MA terms are typically selected manually using the method described by Box and Jenkins [1]. However, the volume of projections required for this study makes manual ARIMA model selection impractical. Instead, we used SAS software's adaption of the United States Census Bureau's X-13ARIMA-SEATS program [2] (SAS software, version 9.4). The X13 procedure includes programming for seasonal adjustments using the X-11-ARIMA procedure [3], and automated model selection using TRAMO (time series regression with ARIMA noise, missing values, and outliers) [4].

The automated model selection procedure is complex; a full description can be found in the US census bureau's reference manual [2]. Briefly, a default model is estimated along with residual diagnostics to be compared with the selected model at a later step. Empirical root tests determine the orders of differencing to make the series stationary, and an iterative process is used to fit multiple models with different AR and MA terms. The best model is selected using BIC and then compared to the default model. The better performing model is retained, and a final model evaluation is performed during which orders of differencing or model terms may be adjusted.

We used the final model for each outcome to create projected monthly rates for 13 months following February 2020. We compared observed monthly rates with projected rates, considering observed rates outside of the projected 95% confidence intervals to be significantly different [5]. Comparison between projected and observed rates are presently graphically as a time series and in tabular form using mean rates across four time periods. We performed all data analyses in SAS software version 9.4 using SAS Enterprise guide version 7.15.3.

**S-References**

1. Box GEP, Jenkins GM, Reinsel GC. Time series analysis: forecasting and control: John Wiley; 2008.

2. Time series research staff, Center for Statistical Research and Methodology, U.S. Census Bureau. Reference Manual for X-13ARIMA-SEATS 2017. Available from: <http://www.census.gov/srd/www/x13as/>.

3. Dagum EB. The X11ARIMA/88 Seasonal Adjustment Method - Foundations and User's Manual. Time Series Research and Analysis Division. . 1988.

4. Gomez V, Maravall A. Automatic modeling methods for univariate series. In: Pena D, Tiao GC, Tsay RS, editors. A Course in Time Series Analysis. New York, NY: J. Wiley and Sons; 2001.

5. Huang YT, Lee YC, Hsiao CJ. Hospitalization for ambulatory-care-sensitive conditions in Taiwan following the SARS outbreak: a population-based interrupted time series study. J Formos Med Assoc. 2009;108(5):386-94. Epub 2009/05/16. doi: 10.1016/S0929-6646(09)60082-6. PubMed PMID: 19443292; PubMed Central PMCID: PMCPMC7135451.
