# Supplementary material for: Healthcare utilization trends in adults with asthma or COPD during the first year of COVID-19 pandemic in comparison to pre-pandemic: A population-based study
Source: PLoS One. 2025 Mar 6;20(3):e0316553. doi: 10.1371/journal.pone.0316553 (PMC11884700; doi:10.1371/journal.pone.0316553)
Supplement: S1 Table — (DOCX) [file pone.0316553.s004.docx]

**S1 Table. Definitions to derive the population of interest and variables at baseline from health administrative databases.**

| **Conditions/variables** | **ICD 9/ICD 10 CA code range** | **OHIP diagnostic codes** |
| --- | --- | --- |
| Definition of physician-diagnosed asthma* | Inpatient/SDS: any diagnostic code in J45, J46  It consists of one asthma hospitalization or two outpatient asthma visits within two years: 84% sensitivity and 76% specificity in adults when compared to a clinical reference standard which, as seen in real-world practice, may or may not have included spirometry.[1-3] | 493 |
| Definition of physician-diagnosed COPD* | Inpatient/SDS: any diagnostic code in J41, J42, J43, J44  It consists of one COPD hospitalization or outpatient visit after 35 years of age [4]: 85% sensitivity and 78% specificity when compared with clinical evaluation.[4-7] | 491, 492, 496 |
|  |  | |
| Baseline demographics (from RPDB; from the Ontario Census) | - Age, sex (from RPDB) - Rural status: Rural residence vs urban was assigned based on the patient's postal code at the time of the index month.[8] The postal code is linked to a binary rurality indicator based on the Ontario Census data. The indicator is based on community size with a size ≤ 10,000 people being classified as rural. - Income status: Ontario neighbourhoods are classified into one of the five approximately equal-sized income quintiles, ranked from poorest (Q1) to wealthiest (Q5), and these have been shown to be related to population health status and health care utilization.[9] Each patient was assigned to the income quintile based on the patient's postal code at the time of index date and Statistics Canada's Postal Code Conversion File.[10, 11] | |

*Prevalent (at any previous time points) and with a healthcare encounter for their condition within the last 5 years prior to the index month.

ICD, International Classification of Diseases; OHIP, Ontario Health Insurance Plan; RPDB, the Registered Persons Database; SDS, same-day-surgery

**S References**

1. Gershon A WC, Vasilevska-Ristovska J, Guan J, Cicutto L, To T. Identifying patients diagnosed with asthma using health administrative data. Canadian Respiratory Journal. 2009;16:183–8.

2. To T, Wang C, Guan J, McLimont S, Gershon AS. What is the lifetime risk of physician-diagnosed asthma in Ontario, Canada? American journal of respiratory and critical care medicine. 2010;181(4):337-43. Epub 2009/11/21. doi: 10.1164/rccm.200907-1035OC. PubMed PMID: 19926867.

3. Gershon AS, Guan J, Wang C, To T. Trends in asthma prevalence and incidence in Ontario, Canada, 1996-2005: a population study. American journal of epidemiology. 2010;172(6):728-36. Epub 2010/08/19. doi: 10.1093/aje/kwq189. PubMed PMID: 20716702.

4. Gershon AS, Wang C, Guan J, Vasilevska-Ristovska J, Cicutto L, To T. Identifying individuals with physcian diagnosed COPD in health administrative databases. Copd. 2009;6(5):388-94. Epub 2009/10/30. PubMed PMID: 19863368.

5. Gershon AS, Campitelli MA, Croxford R, Stanbrook MB, To T, Upshur R, et al. Combination long-acting beta-agonists and inhaled corticosteroids compared with long-acting beta-agonists alone in older adults with chronic obstructive pulmonary disease. Jama. 2014;312(11):1114-21. Epub 2014/09/17. doi: 10.1001/jama.2014.11432. PubMed PMID: 25226477.

6. Gershon AS, Warner L, Cascagnette P, Victor JC, To T. Lifetime risk of developing chronic obstructive pulmonary disease: a longitudinal population study. Lancet. 2011;378(9795):991-6. Epub 2011/09/13. doi: 10.1016/S0140-6736(11)60990-2. PubMed PMID: 21907862.

7. Gershon AS, Wang C, Wilton AS, Raut R, To T. Trends in chronic obstructive pulmonary disease prevalence, incidence, and mortality in ontario, Canada, 1996 to 2007: a population-based study. Archives of internal medicine. 2010;170(6):560-5. Epub 2010/03/24. doi: 10.1001/archinternmed.2010.17. PubMed PMID: 20308643.

8. Ontario Ministry of Health and Long-Term Care. Health Analyst’s Toolkit. Available from: <http://www.health.gov.on.ca/english/providers/pub/healthanalytics/health_toolkit/health_toolkit.pdf> 2012.

9. Wilkins R, Tjepkema M, Mustard C, Choiniere R. The Canadian census mortality follow-up study, 1991 through 2001. Health reports / Statistics Canada, Canadian Centre for Health Information = Rapports sur la sante / Statistique Canada, Centre canadien d'information sur la sante. 2008;19(3):25-43. Epub 2008/10/14. PubMed PMID: 18847143.

10. Wilkins R. Use of postal codes and addresses in the analysis of health data. Health reports / Statistics Canada, Canadian Centre for Health Information = Rapports sur la sante / Statistique Canada, Centre canadien d'information sur la sante. 1993;5(2):157-77. Epub 1993/01/01. PubMed PMID: 8292756.

11. Southern DA, Faris PD, Knudtson ML, Ghali WA. Prognostic relevance of census-derived individual respondent incomes versus household incomes. Canadian journal of public health = Revue canadienne de sante publique. 2006;97(2):114-7. Epub 2006/04/20. PubMed PMID: 16619997.
