# Supplementary material for: Healthcare utilization trends in adults with asthma or COPD during the first year of COVID-19 pandemic in comparison to pre-pandemic: A population-based study
Source: PLoS One. 2025 Mar 6;20(3):e0316553. doi: 10.1371/journal.pone.0316553 (PMC11884700; doi:10.1371/journal.pone.0316553)
Supplement: S2 Table — (A-B). Monthly crude rates, crude rate ratios (RR) and 95% confidence intervals (CI) for all-cause outpatient, inpatient visits, and pulmonary function tests (PFT) in adults with a pre-existing physician diagnosis of asthma or COPD during the first year of the pandemic compared to pre-pandemic. (DOCX) [file pone.0316553.s005.docx]

**S2 Table (A-B). Monthly crude rates, crude rate ratios (RR) and 95% confidence intervals (CI) for all-cause outpatient, inpatient visits, and pulmonary function tests (PFT) in adults with a pre-existing physician diagnosis of asthma during the first year of the pandemic compared to pre-pandemic.**

**S2-A Table**. **Monthly crude rates, crude rate ratios (RR) and 95% confidence intervals (CI) for all-cause outpatient, inpatient visits, and pulmonary function tests (PFT) in adults with a pre-existing physician diagnosis of asthma during the first year of the pandemic compared to pre-pandemic.**

| **Outcomes** | **2017-19** | **2020** | **RR (95%CI)** | **2017-19** | **2020** | **RR (95%CI)** | **2017-19** | **2020** | **RR (95%CI)** | **2017-19** | **2020-21** | **RR (95%CI)** |
| --- | --- | --- | --- | --- | --- | --- | --- | --- | --- | --- | --- | --- |
|  | **Jan-Feb** | |  | **Mar-May** | |  | **Jun-Aug** | |  | **Sep-Mar** | |  |
|  | Monthly rates per 100,000 people | |  | Monthly rates per 100,000 people | |  | Monthly rates per 100,000 people | |  | Monthly rates per 100,000 people | |  |
| Outpatient visits | 73,294.95 | 73,666.5 | 1.01  (0.89-1.14) | 77,694.88 | 64,972.98 | **0.84**  **(0.79-0.89)** | 73,219.88 | 73,568.96 | 1.00  (0.96-1.05) | 74,164.38 | 80,293.12 | **1.08**  **(1.01-1.16)** |
| Primary care visits | 47,839.07 | 47,187.08 | 0.99  (0.86-1.14) | 49,648.07 | 42,982.76 | **0.87**  **(0.81-0.92)** | 46,968.55 | 47,564.86 | 1.01  (0.97-1.05) | 47,809.02 | 50,850.78 | 1.06  (1.00-1.13) |
| Other Specialist visits | 25,456.33 | 26,479.58 | 1.04  (0.94-1.16) | 28,047.06 | 21,990.22 | **0.78**  **(0.73-0.84)** | 26,251.36 | 26,004.21 | 0.99  (0.94-1.04) | 26,355.59 | 29,442.38 | **1.12**  **(1.04-1.20)** |
| Outpatient virtual visits | 889.24 | 1,527.55 | **1.72**  **(1.21-2.45)** | 963.39 | 40,325.37 | **41.86 (29.55-59.30)** | 1,018.42 | 45,119.81 | **44.30**  **(33.76-58.14)** | 2,239.8 | 47,239.94 | **21.09 (10.00-44.48)** |
| ED visits | 6,279.21 | 6,171.13 | 0.98  (0.88-1.09) | 6,524.84 | 4,312.75 | **0.66**  **(0.59-0.74)** | 6,606.07 | 5,648.34 | **0.86**  **(0.82-0.89)** | 6,293.44 | 5,027.6 | **0.80**  **(0.75-0.85)** |
| Hospitalizations | 1,142.33 | 1,132.89 | 0.99  (0.90-1.09) | 1,162.05 | 804.23 | **0.69**  **(0.64-0.75)** | 1,102.04 | 971.66 | **0.88**  **(0.85-0.92)** | 1,118.22 | 1,002.53 | **0.90**  **(0.85-0.94)** |
| Pulmonary function tests | 2,330.44 | 2,417.98 | 1.04  (0.92-1.17) | 2,623.56 | 476.34 | **0.18**  **(0.08-0.43)** | 2,447.49 | 688.57 | **0.28**  **(0.22-0.36)** | 2,375.72 | 1,246.68 | **0.52**  **(0.46-0.60)** |

In bold: statistically significant

CI, confidence intervals; ED, emergency department; RR, rate ratios

**S2-B Table. Monthly crude rates, crude rate ratios (RR) and 95% confidence intervals (CI) for all-cause outpatient, inpatient visits, and pulmonary function tests in adults with a pre-existing physician diagnosis of COPD during the first year of the pandemic compared to pre-pandemic.**

| **Outcomes** | **2017-19** | **2020** | **RR (95%CI)** | **2017-19** | **2020** | **RR (95%CI)** | **2017-19** | **2020** | **RR (95%CI)** | **2017-19** | **2020-21** | **RR (95%CI)** |
| --- | --- | --- | --- | --- | --- | --- | --- | --- | --- | --- | --- | --- |
|  | **Jan-Feb** | |  | **Mar-May** | |  | **Jun-Aug** | |  | **Sep-Mar** | |  |
|  | Monthly rates per 100,000 people | |  | Monthly rates per 100,000 people | |  | Monthly rates per 100,000 people | |  | Monthly rates per 100,000 people | |  |
| Outpatient visits | 83985.09 | 84146.22 | 1.00  (0.89-1.13) | 90747.06 | 75975.6 | **0.84**  **(0.79-0.89)** | 85768.93 | 85554.8 | 1.00  (0.95-1.05) | 85889.49 | 92650.52 | 1.08  (1.00-1.16) |
| Primary care visits | 51363.89 | 49851.76 | 0.97  (0.85-1.11) | 54226.38 | 47708.1 | **0.88**  **(0.84-0.92)** | 51690.99 | 52234.31 | 1.01  (0.96-1.06) | 51563.81 | 54964.72 | 1.07  (1.00-1.14) |
| Other Specialist visits | 32621.59 | 34294.45 | 1.05  (0.94-1.17) | 36520.94 | 28267.5 | **0.77**  **(0.71-0.84)** | 34077.96 | 33320.64 | 0.98  (0.92-1.03) | 34325.86 | 37685.83 | **1.10**  **(1.02-1.19)** |
| Outpatient virtual visits | 1139.21 | 1749.21 | **1.54**  **(1.12-2.10)** | 1248.61 | 44469.79 | **35.62 (25.31-50.13)** | 1304.51 | 48176.02 | **36.93 (28.69-47.53)** | 2556.35 | 49419.48 | **19.33**  **(9.66-38.70)** |
| ED visits | 8682.55 | 8474.9 | 0.98  (0.87-1.09) | 9104.63 | 6169.9 | **0.68**  **(0.62-0.75)** | 9198.31 | 7989.28 | **0.87**  **(0.84-0.90)** | 8673.07 | 7040.58 | **0.81**  **(0.76-0.86)** |
| Hospitalizations | 2729.82 | 2720.31 | 1.00  (0.90-1.11) | 2717.35 | 1951.03 | **0.72**  **(0.67-0.77)** | 2530.91 | 2278.92 | **0.90**  **(0.87-0.93)** | 2636.08 | 2326.07 | **0.88**  **(0.84-0.93)** |
| Pulmonary function tests | 3141.03 | 3149.44 | 1.00  (0.90-1.12) | 3597.56 | 678.6 | **0.19**  **(0.09-0.41)** | 3317.82 | 1016.3 | **0.31**  **(0.24-0.39)** | 3196.23 | 1703.33 | **0.53**  **(0.47-0.61)** |

In bold: statistically significant

CI, confidence intervals; ED, emergency department; RR, rate ratios
