# Supplementary material for: Healthcare utilization trends in adults with asthma or COPD during the first year of COVID-19 pandemic in comparison to pre-pandemic: A population-based study
Source: PLoS One. 2025 Mar 6;20(3):e0316553. doi: 10.1371/journal.pone.0316553 (PMC11884700; doi:10.1371/journal.pone.0316553)
Supplement: S3 Table — (A-B). Observed and projected monthly rates and 95% confidence intervals (CI) estimated by ARIMA Models for all-cause hospitalizations, emergency department (ED) and outpatient visits in adults with a pre-existing physician diagnosis of asthma or COPD: rates were calculated as the number of events per 100,000 people at risk. Similar periods in previous years (2016-2019) were used to calculate projected rates. (DOCX) [file pone.0316553.s006.docx]

**S3 Table (A-B).** **Observed and projected monthly rates and 95% confidence intervals (CI) estimated by ARIMA Models for all-cause hospitalizations, emergency department (ED) and outpatient visits in adults with a pre-existing physician diagnosis of asthma or COPD: rates were calculated as the number of events per 100,000 people at risk. Similar periods in previous years (2016-2019) were used to calculate projected rates.**

**S3-A Table.** **Observed and projected monthly rates and 95% confidence intervals (CI) estimated by ARIMA Models for all-cause hospitalizations, emergency department (ED) and outpatient visits in adults with a pre-existing physician diagnosis of asthma: rates were calculated as the number of events per 100,000 people at risk. Similar periods in previous years (2016-2019) were used to calculate projected rates.**

| **Entire population** | **Observed** | **Projected**  **(95% CI)** | **Observed** | **Projected**  **(95% CI)** | **Observed** | **Projected**  **(95% CI)** | **Observed** | **Projected**  **(95% CI)** |
| --- | --- | --- | --- | --- | --- | --- | --- | --- |
|  | Jan-Feb 2020 | | Mar-May 2020 | | Jun-Aug 2020 | | Sep 2020 - Mar 2021 | |
|  |  |  |  |  |  |  |  |  |
| All-cause outpatient visits | 73,666.50 | 71,049.60 (66625.55-75767.41) | **64,972.98** | 77191.90 (72095.66-82649.01) | 73,568.96 | 72402.53 (67497.26-77664.28) | **80,293.12** | 74192.24 (68925.50-79867.45) |
| Overall primary care visits | 47,187.08 | 46,273.62 (43225.71-49536.44) | **42,982.76** | 49054.90 (45761.72-52585.11) | 47,564.86 | 46571.02 (43412.65-49959.17) | 50,850.78 | 47636.56 (44286.96-51241.72) |
| Overall specialist visits | 26,479.58 | 25,576.07 (24303.05-26915.79) | **21,990.22** | 28187.76 (26293.11-30223.17) | 26,004.21 | 26436.73 (24464.03-28568.51) | **29,442.38** | 26883.29 (24826.06-29111.80) |
| Outpatient virtual visits | 1,527.55 | 1,505.11 (1419.92-1590.30) | **40,325.37** | 1598.37 (1512.12-1684.61) | **45,119.81** | 1648.43 (1545.76-1751.09) | **47,239.94** | 1786.97 (1642.40-1931.55) |
| All-cause ED visits | 6,171.13 | 6,260.21 (6001.04-6530.58) | **4,312.75** | 6466.67 (6166.02-6782.07) | **5,648.34** | 6559.15 (6237.44-6897.46) | **5,027.60** | 6322.20 (6008.36-6652.45) |
| All-cause hospitalizations | 1,132.89 | 1,137.46 (1084.65-1192.84) | **804.23** | 1163.95 (1109.91-1220.62) | **971.66** | 1093.17 (1042.41-1146.39) | **1,002.53** | 1126.99 (1073.44-1183.21) |
| Pulmonary function tests | 2,417.98 | 2,445.84 (2266.11-2639.83) | **476.34** | 2733.74 (2510.79-2976.60) | **688.57** | 2480.53 (2266.93-2714.26) | **1,246.68** | 2526.03 (2298.24-2776.68) |

In bold: statistically significant

CI, confidence intervals; ED, emergency department

**S3-B Table.** **Observed and projected monthly rates and 95% confidence intervals (CI) estimated by ARIMA Models for all-cause hospitalizations, emergency department (ED) and outpatient visits in adults with a pre-existing physician diagnosis of COPD: rates were calculated as the number of events per 100,000 people at risk. Similar periods in previous years (2016-2019) were used to calculate projected rates.**

| **Entire population** | **Observed** | **Projected**  **(95% CI)** | **Observed** | **Projected**  **(95% CI)** | **Observed** | **Projected**  **(95% CI)** | **Observed** | **Projected**  **(95% CI)** |
| --- | --- | --- | --- | --- | --- | --- | --- | --- |
|  | Jan-Feb 2020 | | Mar-May 2020 | | Jun-Aug 2020 | | Sep 2020 - Mar 2021 | |
|  |  |  |  |  |  |  |  |  |
| All-cause outpatient visits | 84,146.22 | 81,583.34 (76603.80-86886.57) | **75,975.60** | 90071.23 (84299.49-96238.60) | 85,554.80 | 84767.84 (79219.44-90704.85) | **92,650.52** | 85870.52 (79975.40-92206.98) |
| Overall primary care visits | 49,851.76 | 48351.52 (45306.02-51601.74) | **47,708.10** | 52566.09 (49179.94-56185.45) | 52,234.31 | 49901.43 (46647.02-53382.89) | **54,964.72** | 50134.19 (46686.83-53840.93) |
| Overall specialist visits | 34,294.45 | 32691.26 (30886.24-34601.77) | **28,267.50** | 36750.63 (34159.75-39542.16) | 33,320.64 | 34562.83 (31914.83-37430.53) | 37,685.83 | 35165.16 (32393.23-38175.76) |
| Outpatient virtual visits | 1,749.21 | 1727.33 (1631.97-1822.69) | **44,469.79** | 1864.48 (1766.74-1962.22) | **48,176.02** | 1912.89 (1790.31-2035.46) | **49,419.48** | 2012.97 (1856.24-2169.71) |
| All-cause ED visits | 8,474.90 | 8588.39 (8219.46-8973.88) | **6,169.90** | 9111.53 (8720.13-9520.50) | **7,989.28** | 9186.97 (8792.33-9599.32) | **7,040.58** | 8763.98 (8382.07-9163.32) |
| All-cause hospitalizations | 2,720.31 | 2676.59 (2560.61-2797.83) | **1,951.03** | 2737.74 (2619.01-2861.84) | **2,278.92** | 2550.33 (2439.69-2665.99) | **2,326.07** | 2667.80 (2545.31-2796.30) |
| Pulmonary function tests | 3,149.44 | 3260.21 (2942.02-3612.81) | **678.60** | 3774.48 (3405.99-4182.83) | **1,016.30** | 3300.65 (2978.38-3657.79) | **1,703.33** | 3354.51 (3007.02-3743.11) |

In bold: statistically significant

CI, confidence intervals; ED, emergency department
