# Supplementary material for: Healthcare utilization trends in adults with asthma or COPD during the first year of COVID-19 pandemic in comparison to pre-pandemic: A population-based study
Source: PLoS One. 2025 Mar 6;20(3):e0316553. doi: 10.1371/journal.pone.0316553 (PMC11884700; doi:10.1371/journal.pone.0316553)
Supplement: S4 Table — (A-D). Observed and projected monthly rates and 95% confidence intervals (CI) estimated by ARIMA Models for all-cause hospitalizations, emergency department (ED) and outpatient visits in adults with a pre-existing physician diagnosis of asthma (total and stratified by sex and age): rates were calculated as the number of events per 100,000 people at risk. Similar periods in previous years (2016-2019) were used to calculate projected rates. (DOCX) [file pone.0316553.s007.docx]

**S4 Table (A-D).** **Observed and projected monthly rates and 95% confidence intervals (CI) estimated by ARIMA Models for all-cause hospitalizations, emergency department (ED) and outpatient visits in adults with a pre-existing physician diagnosis of asthma (total and stratified by sex and age): rates were calculated as the number of events per 100,000 people at risk. Similar periods in previous years (2016-2019) were used to calculate projected rates.**

**S4-A Table. Observed and projected monthly rates and 95% confidence intervals (CI) estimated by ARIMA Models for outpatient care visits in adults with a pre-existing physician diagnosis of asthma (total and stratified by sex and age): rates were calculated as the number of events per 100,000 people at risk. Similar periods in previous years (2016-2019) were used to calculate projected rates**.

|  | **Observed** | **Projected**  **(95% CI)** | **Observed** | **Projected**  **(95% CI)** | **Observed** | **Projected**  **(95% CI)** | **Observed** | **Projected**  **(95% CI)** |
| --- | --- | --- | --- | --- | --- | --- | --- | --- |
|  | Jan-Feb 2020 | | Mar-May 2020 | | Jun-Aug 2020 | | Sep 2020 - Mar 2021 | |
| **All-cause outpatient visits** | | | | | | | | |
| Total | 73666.50 | 71049.60 (66625.55-75767.41) | **64972.98** | 77191.90 (72095.66-82649.01) | 73568.96 | 72402.53 (67497.26-77664.28) | **80293.12** | 74192.24 (68925.50-79867.45) |
| Age 18-24 | 43206.52 | 42263.53 (39597.18-45109.42) | **35635.84** | 43883.22 (40972.96-47000.49) | 42453.04 | 43156.47 (40142.83-46396.35) | 45284.82 | 42494.45 (39431.16-45796.66) |
| Age 25-34 | 55801.20 | 53929.89 (50555.38-57529.66) | **49529.65** | 56516.39 (52840.12-60448.66) | 54846.72 | 53492.07 (49874.87-57371.61) | **60034.57** | 54916.62 (51066.01-59059.66) |
| Age 35-49 | 66320.33 | 63919.04 (59868.48-68243.65) | **58908.00** | 66954.85 (62419.31-71820.74) | 63676.17 | 61252.33 (56915.24-65919.92) | **70685.71** | 64507.93 (59722.17-69682.79) |
| Age 50-64 | 79954.39 | 76295.96 (71343.79-81591.87) | **70830.10** | 82949.87 (77317.63-88992.84) | 78400.02 | 76574.09 (71231.06-82317.89) | **85639.87** | 79260.45 (73446.85-85541.95) |
| Age 65+ | 98517.37 | 93956.41 (87649.41-100717.24) | **87117.05** | 108164.45 (100609.84-116286.77) | 102844.10 | 102148.21 (94855.32-110001.80) | **110814.78** | 102331.96 (94659.93-110636.35) |
| Sex: female | 80599.53 | 77626.18 (72694.49-82892.44) | **71819.87** | 84899.60 (79153.82-91063.28) | 81755.92 | 79450.25 (73921.96-85391.98) | **89245.48** | 81333.33 (75406.77-87732.53) |
| Sex: male | 63479.92 | 61100.31 (57408.68-65029.32) | **54909.80** | 65904.73 (61657.87-70444.71) | 61519.78 | 62017.37 (57861.90-66471.28) | 67140.40 | 63691.26 (59225.53-68498.42) |

| **Overall primary care visits** | | | | | | | | |
| --- | --- | --- | --- | --- | --- | --- | --- | --- |
| Total | 47187.08 | 46273.62 (43225.71-49536.44) | **42982.76** | 49054.90 (45761.72-52585.11) | 47564.86 | 46571.02 (43412.65-49959.17) | 50850.78 | 47636.56 (44286.96-51241.72) |
| Age 18-24 | 31822.20 | 30810.31 (28616.67-33172.11) | **25389.95** | 31940.71 (29511.48-34570.39) | 30317.23 | 31585.87 (29075.16-34313.39) | 31960.98 | 31475.84 (28926.22-34250.72) |
| Age 25-34 | 38344.93 | 36740.47 (34356.66-39289.67) | **34079.23** | 38252.87 (35717.19-40968.60) | 37076.32 | 36441.37 (33972.40-39089.77) | 40067.41 | 37679.18 (34996.86-40568.95) |
| Age 35-49 | 45107.25 | 43279.84 (40272.05-46512.28) | **40708.91** | 45011.08 (41759.51-48516.04) | 42802.90 | 41189.94 (38144.12-44478.98) | 46978.89 | 43639.15 (40245.49-47323.96) |
| Age 50-64 | 51026.96 | 49491.55 (46177.54-53043.39) | **46986.67** | 52894.47 (49301.73-56749.07) | 50934.95 | 49565.55 (46100.57-53290.95) | 54248.15 | 51094.13 (47376.24-55106.55) |
| Age 65+ | 57484.61 | 55251.58 (51360.60-59437.32) | **54328.87** | 62450.06 (58013.54-67225.88) | 62508.13 | 59574.91 (55323.45-64153.08) | **65553.70** | 59355.03 (54882.88-64199.06) |
| Sex: female | 51442.15 | 49201.22 (45737.97-52926.70) | **47461.19** | 53457.13 (49606.62-57606.61) | 52829.30 | 50028.46 (46378.11-53966.13) | **56553.98** | 51436.57 (47479.31-55729.99) |
| Sex: male | 40935.19 | 40219.16 (37704.64-42901.37) | **36400.63** | 42111.21 (39421.73-44984.22) | 39816.90 | 40255.79 (37655.07-43036.13) | 42471.70 | 41079.31 (38330.44-44026.94) |

| **Overall specialist visits** | | | | | | | | |
| --- | --- | --- | --- | --- | --- | --- | --- | --- |
| Total | 26479.58 | 25576.07 (24303.05-26915.79) | **21990.22** | 28187.76 (26293.11-30223.17) | 26004.21 | 26436.73 (24464.03-28568.51) | **29442.38** | 26883.29 (24826.06-29111.80) |
| Age 18-24 | 11385.02 | 11232.18 (10383.80-12149.87) | **10245.89** | 11665.68 (10765.04-12641.72) | 12136.28 | 11503.88 (10547.23-12547.31) | **13323.83** | 11082.11 (10115.47-12141.73) |
| Age 25-34 | 17456.27 | 16979.18 (15748.32-18306.23) | **15450.42** | 18047.01 (16673.37-19533.96) | 17770.39 | 17008.05 (15676.80-18452.33) | **19967.16** | 17161.60 (15752.43-18698.81) |
| Age 35-49 | 21213.08 | 20905.06 (19599.94-22297.08) | **18199.09** | 21940.55 (20456.94-23532.09) | 20873.26 | 20027.91 (18625.82-21535.55) | **23706.81** | 20877.74 (19349.47-22528.37) |
| Age 50-64 | 28927.75 | 28098.34 (26609.15-29670.87) | **23843.43** | 31247.58 (29269.88-33360.68) | 27465.07 | 28147.32 (26067.21-30393.43) | 31391.71 | 29113.11 (26896.64-31513.37) |
| Age 65+ | 41032.76 | 38484.52 (36063.23-41068.37) | **32788.18** | 45026.04 (41726.93-48588.39) | 40336.18 | 42434.83 (39150.44-45994.74) | 45261.26 | 42651.58 (39235.35-46368.01) |
| Sex: female | 29157.66 | 28499.86 (27113.01-29957.65) | **24358.68** | 31341.55 (29257.90-33578.63) | 28926.71 | 29342.66 (27164.27-31695.73) | **32691.57** | 29684.45 (27422.45-32133.89) |
| Sex: male | 22544.72 | 21931.24 (20853.81-23064.33) | **18509.17** | 24223.51 (22650.21-25909.42) | 21703.01 | 22363.81 (20668.71-24197.92) | 24668.70 | 23033.60 (21241.65-24977.42) |

**S4-B Table. Observed and projected monthly rates and 95% confidence intervals (CI) estimated by ARIMA Models for all-cause emergency department (ED) visits in adults with a pre-existing physician diagnosis of asthma (total and stratified by sex and age): rates were calculated as the number of events per 100,000 people at risk. Similar periods in previous years (2016-2019) were used to calculate projected rates.**

|  | **Observed** | **Projected**  **(95% CI)** | **Observed** | **Projected**  **(95% CI)** | **Observed** | **Projected**  **(95% CI)** | **Observed** | **Projected**  **(95% CI)** |
| --- | --- | --- | --- | --- | --- | --- | --- | --- |
|  | Jan-Feb 2020 | | Mar-May 2020 | | Jun-Aug 2020 | | Sep 2020 - Mar 2021 | |
| Total | 6171.13 | 6260.21  (6001.04-6530.58) | **4312.75** | 6466.67  (6166.02-6782.07) | **5648.34** | 6559.15  (6237.44-6897.46) | **5027.60** | 6322.20  (6008.36-6652.45) |
| Age 18-24 | 6254.19 | 6264.17  (5937.29-6609.05) | **3951.99** | 6434.19  (6098.35-6788.51) | **5628.35** | 6719.37  (6368.65-7089.40) | **4768.99** | 6450.79  (6107.88-6812.99) |
| Age 25-34 | 6508.61 | 6277.42  (5945.37-6628.01) | **4520.88** | 6433.46  (6088.19-6798.32) | **5826.60** | 6548.92  (6197.45-6920.33) | **5275.79** | 6376.87  (6031.09-6742.49) |
| Age 35-49 | 5558.99 | 5577.11  (5242.49-5933.09) | **4026.44** | 5765.75  (5415.26-6138.91) | **5106.26** | 5750.44  (5387.46-6137.87) | **4692.12** | 5655.77  (5273.54-6065.77) |
| Age 50-64 | 5541.47 | 5615.49  (5338.07-5907.31) | **4062.56** | 5856.79  (5567.41-6161.21) | **5171.45** | 5841.95  (5553.30-6145.59) | **4528.13** | 5677.40  (5391.23-5978.80) |
| Age 65+ | 7123.17 | 7104.38  (6698.21-7535.18) | **4865.09** | 7776.24  (7265.51-8323.20) | **6521.42** | 7830.63  (7284.39-8417.84) | **5793.70** | 7207.45  (6682.66-7773.95) |
| Sex: female | 6678.35 | 6734.68  (6430.78-7052.94) | **4578.05** | 7057.15  (6738.70-7390.65) | **5946.47** | 7006.87  (6690.68-7337.99) | **5345.10** | 6848.48  (6533.63-7178.53) |
| Sex: male | 5425.87 | 5420.23  (5177.24-5674.64) | **3922.86** | 5676.72  (5420.36-5945.20) | **5209.56** | 5827.88  (5559.05-6109.71) | **4561.13** | 5527.20  (5260.43-5807.52) |

**S4-C Table. Observed and projected monthly rates and 95% confidence intervals (CI) estimated by ARIMA Models for all-cause hospitalizations in adults with a pre-existing physician diagnosis of asthma (total and stratified by sex and age): rates were calculated as the number of events per 100,000 people at risk. Similar periods in previous years (2016-2019) were used to calculate projected rates.**

|  | **Observed** | **Projected**  **(95% CI)** | **Observed** | **Projected**  **(95% CI)** | **Observed** | **Projected**  **(95% CI)** | **Observed** | **Projected**  **(95% CI)** |
| --- | --- | --- | --- | --- | --- | --- | --- | --- |
|  | Jan-Feb 2020 | | Mar-May 2020 | | Jun-Aug 2020 | | Sep 2020 - Mar 2021 | |
| Total | 1132.89 | 1137.46  (1084.65-1192.84) | **804.23** | 1163.95  (1109.91-1220.62) | **971.66** | 1093.17  (1042.41-1146.39) | **1002.53** | 1126.99  (1073.44-1183.21) |
| Age 18-24 | 364.52 | 337.20 (296.49-383.50) | **295.87** | 377.63  (329.31-433.06) | 326.70 | 360.74  (313.26-415.42) | **306.42** | 354.62  (307.52-408.94) |
| Age 25-34 | 694.65 | 693.12  (629.61-763.03) | **624.75** | 715.24  (648.96-788.29) | 689.31 | 742.80  (673.97-818.67) | 676.35 | 697.89  (632.59-769.93) |
| Age 35-49 | 601.65 | 622.96  (576.45-673.22) | **437.07** | 614.52  (568.62-664.12) | **558.34** | 610.17  (564.60-659.42) | 565.74 | 599.66  (553.31-649.93) |
| Age 50-64 | 965.73 | 957.52  (879.37-1042.62) | **660.86** | 978.45  (898.59-1065.41) | **816.28** | 927.00  (851.33-1009.38) | **847.49** | 949.72  (870.56-1036.09) |
| Age 65+ | 2395.53 | 2379.39  (2245.92-2520.80) | **1619.49** | 2463.56  (2320.77-2615.14) | **1961.67** | 2248.22  (2106.12-2399.91) | **2044.88** | 2334.46  (2162.07-2520.83) |
| Sex: female | 1226.08 | 1226.97  (1162.31-1295.23) | **864.75** | 1257.68  (1191.40-1327.64) | **1047.62** | 1174.76  (1112.85-1240.12) | **1083.03** | 1213.71  (1148.05-1283.15) |
| Sex: male | 995.98 | 990.18  (931.07-1053.05) | **715.29** | 1013.21  (949.95-1080.68) | **859.88** | 963.56  (901.77-1029.58) | **884.24** | 989.86  (925.51-1058.69) |

**S4-D Table. Observed and projected monthly rates and 95% confidence intervals (CI) estimated by ARIMA Models for pulmonary function tests in adults with a pre-existing physician diagnosis of asthma (total and stratified by sex and age): rates were calculated as the number of events per 100,000 people at risk. Similar periods in previous years (2016-2019) were used to calculate projected rates.**

|  | **Observed** | **Projected**  **(95% CI)** | **Observed** | **Projected**  **(95% CI)** | **Observed** | **Projected**  **(95% CI)** | **Observed** | **Projected**  **(95% CI)** |
| --- | --- | --- | --- | --- | --- | --- | --- | --- |
|  | Jan-Feb 2020 | | Mar-May 2020 | | Jun-Aug 2020 | | Sep 2020 - Mar 2021 | |
| Total | 2417.98 | 2445.84  (2266.11-2639.83) | **476.34** | 2733.74  (2510.79-2976.60) | **688.57** | 2480.53  (2266.93-2714.26) | **1246.68** | 2526.03  (2298.24-2776.68) |
| Age 18-24 | 958.68 | 953.88  (831.02-1094.91) | **181.15** | 970.16  (845.14-1113.66) | **320.47** | 1051.50  (916.01-1207.04) | **530.13** | 951.23  (826.82-1094.38) |
| Age 25-34 | 1253.68 | 1204.85  (1072.63-1337.07) | **242.98** | 1214.62  (1074.81-1354.42) | **377.92** | 1161.11  (1015.76-1306.46) | **720.94** | 1178.92  (1027.25-1330.60) |
| Age 35-49 | 1838.74 | 1830.38  (1650.78-2029.51) | **380.91** | 1879.35  (1684.58-2096.68) | **542.81** | 1699.48  (1517.32-1903.52) | **991.07** | 1791.02  (1589.73-2018.23) |
| Age 50-64 | 2845.76 | 2866.27  (2639.29-3112.77) | **567.06** | 3173.45  (2908.55-3462.51) | **794.41** | 2816.67  (2573.91-3082.33) | **1474.72** | 2923.69  (2658.80-3215.42) |
| Age 65+ | 3866.40 | 3947.43  (3612.42-4313.51) | **743.47** | 4843.76  (4420.07-5308.08) | **1064.27** | 4284.90  (3904.40-4702.48) | **1869.85** | 4262.27  (3863.64-4702.81) |
| Sex: female | 2453.11 | 2440.76  (2263.33-2632.10) | **481.12** | 2787.60  (2555.73-3040.67) | **696.46** | 2511.27  (2290.09-2753.81) | **1267.01** | 2564.68  (2327.84-2825.92) |
| Sex: male | 2366.36 | 2426.43  (2221.97-2649.71) | **469.32** | 2609.35  (2373.61-2868.57) | **676.95** | 2405.66  (2177.57-2657.65) | **1216.78** | 2437.84  (2194.50-2708.56) |
