# Supplementary material for: Healthcare utilization trends in adults with asthma or COPD during the first year of COVID-19 pandemic in comparison to pre-pandemic: A population-based study
Source: PLoS One. 2025 Mar 6;20(3):e0316553. doi: 10.1371/journal.pone.0316553 (PMC11884700; doi:10.1371/journal.pone.0316553)
Supplement: S5 Table — (A-D). Observed and projected monthly rates and 95% confidence intervals (CI) estimated by ARIMA Models for all-cause hospitalizations, emergency department (ED) and outpatient visits in adults with a pre-existing physician diagnosis of COPD (total and stratified by sex and age): rates were calculated as the number of events per 100,000 people at risk. Similar periods in previous years (2016-2019) were used to calculate projected rates. (DOCX) [file pone.0316553.s008.docx]

**S6 Table (A-B).** **A sensitivity analysis: Observed and projected monthly rates and 95% confidence intervals (CI) estimated by ARIMA Models for all-cause hospitalizations, emergency department (ED) and outpatient visits in adults with a pre-existing physician diagnosis of asthma or COPD: rates were calculated as the number of events per 100,000 people at risk. Similar periods in previous years (2016-2019) were used to calculate projected rates**. As a sensitivity analysis, we limited the automated model procedure to first-order terms for both the non-seasonal and seasonal term values and re-calculated projected values.

**S6-A Table.** **A sensitivity analysis: Observed and projected monthly rates and 95% confidence intervals (CI) estimated by ARIMA Models for all-cause hospitalizations, emergency department (ED) and outpatient visits in adults with a pre-existing physician diagnosis of asthma: rates were calculated as the number of events per 100,000 people at risk. Similar periods in previous years (2016-2019) were used to calculate projected rates.**

| **Entire population** | **Observed** | **Projected**  **(95% CI)** | **Observed** | **Projected**  **(95% CI)** | **Observed** | **Projected**  **(95% CI)** | **Observed** | **Projected**  **(95% CI)** |
| --- | --- | --- | --- | --- | --- | --- | --- | --- |
|  | Jan-Feb 2020 | | Mar-May 2020 | | Jun-Aug 2020 | | Sep 2020 - Mar 2021 | |
| All-cause outpatient visits | 73666.50 | 71049.60 (66625.55- 75767.41) | **64972.98** | 77191.90 (72095.66- 82649.01) | 73568.96 | 72402.53 (67497.26- 77664.28) | **80293.12** | 74192.24 (68925.50- 79867.45) |
| Overall primary care visits | 47187.08 | 46273.59 (43225.70-  49536.38) | **42982.76** | 49054.91 (45761.75- 52585.10) | 47564.86 | 46571.00 (43412.64- 49959.13) | 50850.78 | 47636.55 (44286.96- 51241.70) |
| Overall specialist visits | 26479.58 | 25544.14 (24305.21-  26846.22) | **21990.22** | 28289.83 (26445.16- 30267.03) | 26004.21 | 26316.60 (24394.08- 28390.63) | **29442.38** | 26889.98 (24854.19- 29093.84) |
| Outpatient virtual visits | 1527.55 | 1492.66 (1412.53-  1572.78) | **40325.37** | 1580.08 (1499.39-  1660.77) | **45119.81** | 1619.67 (1526.48- 1712.86) | **47239.94** | 1761.51 (1646.45-  1876.57) |
| All-cause ED visits | 6171.13 | 6260.21 (6001.04-  6530.58) | **4312.75** | 6466.67 (6166.02-  6782.07) | **5648.34** | 6559.15 (6237.44- 6897.46) | **5027.60** | 6322.20 (6008.36- 6652.45) |
| All-cause hospitalizations | 1132.89 | 1137.46 (1084.65-  1192.84) | **804.23** | 1163.95 (1109.91-  1220.62) | **971.66** | 1093.17 (1042.41- 1146.39) | **1002.53** | 1126.99 (1073.44-  1183.21) |
| Pulmonary function tests | 2417.98 | 2451.77 (2262.39-  2657.01) | **476.34** | 2760.30 (2532.02-  3009.21) | **688.57** | 2490.02 (2275.25- 2725.06) | **1246.68** | 2535.04 (2305.87- 2787.33) |

In bold: statistically significant

CI, confidence intervals; ED, emergency department

**S6-B Table.** **A sensitivity analysis: Observed and projected monthly rates and 95% confidence intervals (CI) estimated by ARIMA Models for all-cause hospitalizations, emergency department (ED) and outpatient visits in adults with a pre-existing physician diagnosis of COPD: rates were calculated as the number of events per 100,000 people at risk. Similar periods in previous years (2016-2019) were used to calculate projected rates.**

| **Entire population** | **Observed** | **Projected**  **(95% CI)** | **Observed** | **Projected**  **(95% CI)** | **Observed** | **Projected**  **(95% CI)** | **Observed** | **Projected**  **(95% CI)** |
| --- | --- | --- | --- | --- | --- | --- | --- | --- |
|  | Jan-Feb 2020 | | Mar-May 2020 | | Jun-Aug 2020 | | Sep 2020 - Mar 2021 | |
| All-cause outpatient visits | 84146.22 | 81583.34 (76603.80-  86886.57) | **75975.60** | 90071.23 (84299.49- 96238.60) | 85554.80 | 84767.84 (79219.44-  90704.85) | **92650.52** | 85870.52 (79975.40-  92206.98) |
| Overall primary care visits | 49851.76 | 48351.52 (45306.02-  51601.74) | **47708.10** | 52566.09 (49179.94-  56185.45) | 52234.31 | 49901.43 (46647.02-  53382.89) | **54964.72** | 50134.19 (46686.83-  53840.93) |
| Overall specialist visits | 34294.45 | 32758.34 (30997.65- 34619.03) | **28267.50** | 37003.85 (34483.17-  39712.44) | 33320.64 | 34414.08 (31803.28-  37239.23) | 37685.83 | 35154.63 (32395.79-  38149.80) |
| Outpatient virtual visits | 1749.21 | 1724.84 (1633.48-  1816.19) | **44469.79** | 1844.05 (1750.81-  1937.29) | **48176.02** | 1890.37 (1779.55-  2001.19) | **49419.48** | 1985.91 (1846.95-  2124.88) |
| All-cause ED visits | 8474.90 | 8588.39 (8219.46-  8973.88) | **6169.90** | 9111.53 (8720.13-  9520.50) | **7989.28** | 9186.97 (8792.33-  9599.32) | **7040.58** | 8763.98 (8382.07-  9163.32) |
| All-cause hospitalizations | 2720.31 | 2676.59 (2560.61-  2797.83) | **1951.03** | 2737.74 (2619.01-  2861.84) | **2278.92** | 2550.33 (2439.69-  2665.99) | **2326.07** | 2667.80 (2545.31- 2796.30) |
| Pulmonary function tests | 3149.44 | 3251.45 (2933.21-  3604.22) | **678.60** | 3772.37 (3403.14-  4181.66) | **1016.30** | 3300.84 (2977.76- 3658.97) | **1703.33** | 3353.72 (3005.43- 3743.33) |

In bold: statistically significant

CI, confidence intervals; ED, emergency department
