# Supplementary material for: Healthcare utilization trends in adults with asthma or COPD during the first year of COVID-19 pandemic in comparison to pre-pandemic: A population-based study
Source: PLoS One. 2025 Mar 6;20(3):e0316553. doi: 10.1371/journal.pone.0316553 (PMC11884700; doi:10.1371/journal.pone.0316553)
Supplement: S7 Table — Similar periods in previous years (2016-2019) were used to calculate projected costs. (DOCX) [file pone.0316553.s010.docx]

**S7 Table**. **Observed and projected costs (total and by subgroups) estimated by ARIMA Models for costs in individuals with a pre-existing physician diagnosis of asthma or COPD: in millions, 2021 adjusted dollars. Similar periods in previous years (2016-2019) were used to calculate projected costs.**

| **Type of cost** | **Observed** | **Projected**  **(95% CI)** | **Observed** | **Projected**  **(95% CI)** | **Observed** | **Projected**  **(95% CI)** | **Observed** | **Projected**  **(95% CI)** |
| --- | --- | --- | --- | --- | --- | --- | --- | --- |
|  | Jan-Feb 2020 | | Mar-May 2020 | | Jun-Aug 2020 | | Sep 2020 - Mar 2021 | |
| **Asthma Population** | | | | | | | | |
| Total | 314.77 | 305.20  (291.42-319.62) | **262.96** | 328.03  (312.80-344.01) | 315.35 | 310.44  (295.31-326.35) | 332.10 | 319.16  (302.78-336.45) |
| Physicians | 76.00 | 75.36  (71.58-79.34) | **57.84** | 81.21  (76.94-85.71) | 73.35 | 76.91  (72.76-81.30) | 78.55 | 77.14  (72.76-81.79) |
| Hospitalizations | 128.57 | 124.99  (119.55-130.67) | **110.49** | 130.63  (124.95-136.57) | **132.95** | 123.79  (118.40-129.41) | **139.58** | 127.52  (121.86-133.44) |
| Medications | 56.19 | 56.94  (54.33-59.67) | **54.31** | 58.92  (55.87-62.15) | 59.63 | 56.53  (52.39-61.00) | 60.10 | 58.92  (53.20-65.26) |
| Home-care | 29.20 | 28.63  (27.75-29.51) | **27.54** | 31.10  (30.11-32.10) | **27.73** | 31.42  (30.05-32.80) | **27.75** | 31.17  (29.28-33.07) |
| Laboratory Investigations | 3.98 | 3.96 (3.40-4.62) | **1.98** | 4.03 (3.36-4.85) | 3.28 | 3.65 (2.81-4.73) | 3.49 | 3.59 (2.53-5.10) |
| **COPD Population** | | | | | | | | |
| Total | 531.64 | 525.87  (505.15-547.44) | **460.74** | 551.73  (529.99-574.36) | 524.21 | 529.81  (508.94-551.54) | 527.02 | 539.07  (517.24-561.82) |
| Physicians | 87.64 | 87.51  (83.08-92.17) | **66.73** | 93.81  (88.92-98.97) | **81.97** | 89.07  (84.35-94.05) | **84.21** | 89.57  (84.57-94.87) |
| Hospitalizations | 250.25 | 250.66  (241.95-259.68) | **219.68** | 256.89  (247.52-266.60) | 251.08 | 243.88  (234.71-253.41) | 251.92 | 250.75  (241.20-260.68) |
| Medications | 86.38 | 87.35  (82.35-92.64) | **81.68** | 92.27  (86.89-97.98) | 89.35 | 88.13  (81.78-94.97) | 88.07 | 91.42  (83.38-100.24) |
| Home-care | 79.87 | 78.91  (77.12-80.71) | **77.34** | 83.76  (81.85-85.67) | **76.58** | 84.88  (82.67-87.08) | **73.76** | 82.52  (80.10-84.94) |
| Laboratory Investigations | 3.59 | 3.62 (3.08-4.24) | **2.01** | 3.64 (3.00-4.43) | 2.98 | 3.29 (2.49-4.35) | 3.00 | 3.22 (2.20-4.71) |

In bold: statistically significant

CI, confidence intervals
